# Arthroscopic Iliac Crest Bone Grafting for Shoulder Glenoid Defect Augmentation Using a “Three‐Pulley Four‐Point Antirotation” Suture Anchor Technique

**DOI:** 10.1002/atn2.70233

**Published:** 2026-08-31

**Authors:** Li Gong, Huiming Hou, Wen Zhou, Shaoyong Fan, Liangshen Hu, Ming Zhou

**Affiliations:** ^1^ Sports Medicine Department Jiangxi University of Traditional Chinese Medicine Affiliated Hongdu Hospital Jiangxi China

## Abstract

Treatment options for severe glenoid bone defects caused by recurrent anterior shoulder instability include autologous coracoid transfer and bone graft reconstruction. The effect of bone‐grafting techniques is satisfactory, and the fixation methods for the bone block include screws or suture buttons. However, problems caused by metal plants and high bone resorption rates are a serious concern. In addition, the current anchor suture fixation technique is prone to unstable fixation and bone block rotation. Therefore, we describe an arthroscopic technique for severe glenoid bone defects that use autologous iliac bone grafting, combined with labrum anchors and pulley knot to fix the bone block, known as the “3‐pulley 4‐point antirotation suture anchor technique.” Four labrum suture anchors are placed on the upper and lower edges of the glenoid defect to form 4 force points (4‐point fixation), and the pulley technique is used for knotting to make the fixation more secure and solve the problem of easy rotation.

## Graphical abstract

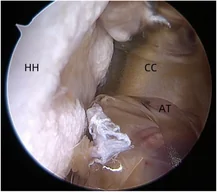

**VIDEO 1:** (1) We will use this video to introduce the iliac bone grafting for shoulder glenoid defect: “3‐pulley 4‐point antirotation” suture anchor technique. (2) First, through the anterior portal, we could observe a significant defect of the anteroinferior glenoid. (3) Use a grinding head to polish the defect of the glenoid bone for freshness. (4) Mark the one‐third and two‐third points along the longitudinal axis of the glenoid bone defect, slightly offset from the midline and corresponding to the superior and inferior lateral edges of it. (5) Through the anterosuperolateral portal, insert the medial inferior labrum anchor. Insert the medial superior labrum anchor. Insert the lateral inferior labrum anchor. Insert the lateral superior labrum anchor. (6) Through the anterosuperolateral portal, insert a head‐removed 10 mL syringe into the glenohumeral joint as a graft channel. (7) The 4 limbs of 2 medial labrum anchors were introduced into the reserved holes of the graft. (8) The bone block was sent into the joint. (9) (1) Make the first pulley knot. Use a wire clamp to pull the 2 limbs of each of the 2 medial labrum anchors out of the joint through anterosuperolateral portal. (2) Make a pulley knot with 2 limbs of each of the 2 medial labrum anchors, and pull it into the joint. (3) Knot the remaining 1 limb of each of the 2 medial labrum anchors together. (10) (1) Make the second pulley knot. Use the wire clamp to pull the remaining limbs of the medial inferior labrum anchor and 2 limbs of the lateral inferior labrum anchor out of the joint through the anterosuperolateral portal. (2) Make a pulley knot with 1 limb of the medial inferior labrum anchor and the lateral inferior labrum anchor, and pull it into the joint. (3) Knot the remaining 1 limb of the medial inferior labrum anchor and the lateral inferior labrum anchor. (11) (1) Make the third pulley knot. Use the wire clamp to pull the remaining limbs of the medial superior labrum anchor and 2 limbs of the lateral superior labrum anchor out of the joint through the anterosuperolateral portal. (2) Make a pulley knot with 1 limb of the medial superior labrum anchor and the lateral superior labrum anchor and pull it into the joint. (3) Knot the remaining 1 limb of the medial superior labrum anchor and the lateral superior labrum anchor. (12) Test the stability of the bone block, very firm. (13) Use the remaining limbs of 2 lateral labrum anchors to repair the labral injury through the anterosuperolateral portal. Video content can be viewed at https://doi.org/10.1002/atn2.70233.

When glenoid bone defects caused by recurrent anterior shoulder dislocation are greater than 25% or more, bony augmentation is required to reduce the postoperative dislocation rate.^1^ It includes coracoid process transposition and bone grafting. The percentage of studies that have reported the average bone resorption rate of the coracoid process transposition surgery are 78%,^2^ and the incidence of postoperative complications is as high as 15% to 30%, including screw loosening, bending, breakage, and neurovascular injuries.^3^, ^4^, ^5^ Moreover, compared with bone transplantation, patients have more significant limitations in shoulder internal rotation and lower functional scores after surgery.^6^ The bone block of transplantation is easy to shape and can meet the needs of different degrees of bone defect repair. Because of the absence of intercepting the coracoid and conjoint tendon, it can effectively avoid damage to the axillary nerve and subscapularis muscle.

In arthroscopic bone grafting, anchor suture fixation can prevent the problems of metal implants and high bone resorption caused by screw and suture button.^7^, ^8^, ^9^ Furthermore, because of its elastic fixation, it causes less damage to bone block and can reduce resorption.^10^, ^11^, ^12^ However, Ritter et al.^13^ have pointed out that the current anchor suture fixation has problems such as insufficient strength and unstable rotation. We therefore describe an arthroscopic reconstruction technique that uses autologous iliac bone grafting with anchor suture fixation using 4 labrum suture anchors combined with pulley knot. Four labrum suture anchors are placed on the upper and lower edges of the glenoid defect, and the bone block is fixed with a pulley knot to enhance stability and reduce bone block absorption. The detailed surgical steps are shown in Video 1. The advantages, disadvantages, and potential complications of this technique are detailed in Table 1.

**TABLE 1:** Advantages and Disadvantages of the Technique

Advantages
• Bone blocks are easily harvested and shaped
• Bone block is stressed evenly and fixed firmly, does not rotate easily
• Avoid the related complications caused by implanted metal
• Avoid damage to the axillary nerve and subscapularis muscle
• No need to drill tunnels, minimal damage to the glenoid bone, and reduced bone resorption
• Easy to operate, reproducible technique, and no need for other special tools
• As a revision procedure of the Latarjet technique

Disadvantages
• Bone block needs to be trimmed
• High precision for the insertion locations of labrum anchors
• Complications of donor site
• Relatively weak maximum failure load compared with the screw

## SURGICAL TECHNIQUE

### Preoperative Assessment

The inclusion criteria for this procedure are as follows: (1) recurrent anterior shoulder instability with a minimum of 3 documented dislocations; (2) a significant glenoid bone loss quantified as ≥25% of the glenoid width on preoperative 3‐dimensional computed tomography; and (3) an active, skeletally mature individual between 18 and 40 years of age with high functional demands for a return to sports or physically demanding activities.

### Patient Setup and Arthroscopic Diagnosis

After the induction of general anesthesia, the patient is positioned supine. A bilateral anterior drawer test is performed to comparatively assess glenohumeral stability. The patient is then transferred to the lateral decubitus position with the unaffected side dependent. The affected extremity is secured in a shoulder traction device (Star, Beijing, China) and maintained in approximately 30° of abduction.

Surgical landmarks for the arthroscopic portals are outlined. A standard sterile preparation and draping is performed to include both the affected shoulder and the ipsilateral iliac crest, the designated autograft harvest site.

The surgical portal is established using standard posterior, anterosuperolateral, and anterior portals. Initially, the posterior portal is created approximately 2 cm inferior and 2 cm medial to the posterolateral corner of the acromion, providing access to the glenohumeral joint. The anterior portal is then established under direct arthroscopic visualization.

**FIGURE 1:**
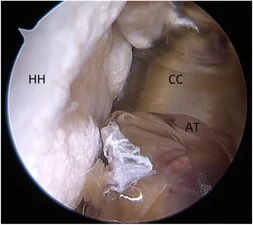
Right shoulder. Arthroscopic view, posterior portal. Hill‐Sachs lesion in the posterosuperior aspect of the humeral head. (AT, annular tubes; CC, capsulolabral complex; HH, humeral head.)

**FIGURE 2:**
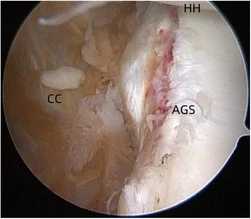
Right shoulder. Arthroscopic view, anterior portal. A significant defect of the anteroinferior glenoid. (AGS, anterior glenoid surface; CC, capsulolabral complex; HH, humeral head.)

Intra‐articular examination reveals a longitudinal bony defect in the posterosuperior aspect of the humeral head (Hill‐Sachs lesion) (Figure 1) through the posterior portal and a significant defect of the anteroinferior glenoid through the anterior portal (Figure 2). The dimensions of the glenoid bone defect, including its length and width, are quantitatively assessed using an arthroscopic measuring probe.

### Preparation of Iliac Crest Graft

**FIGURE 3:**
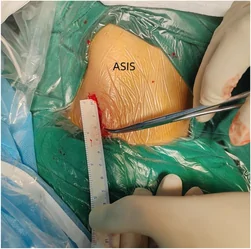
Two‐centimeter oblique longitudinal skin incision on 3 cm posterior to the anterior superior iliac spine of the ipsilateral side. (ASIS, anterior superior iliac spine.)

**FIGURE 4:**
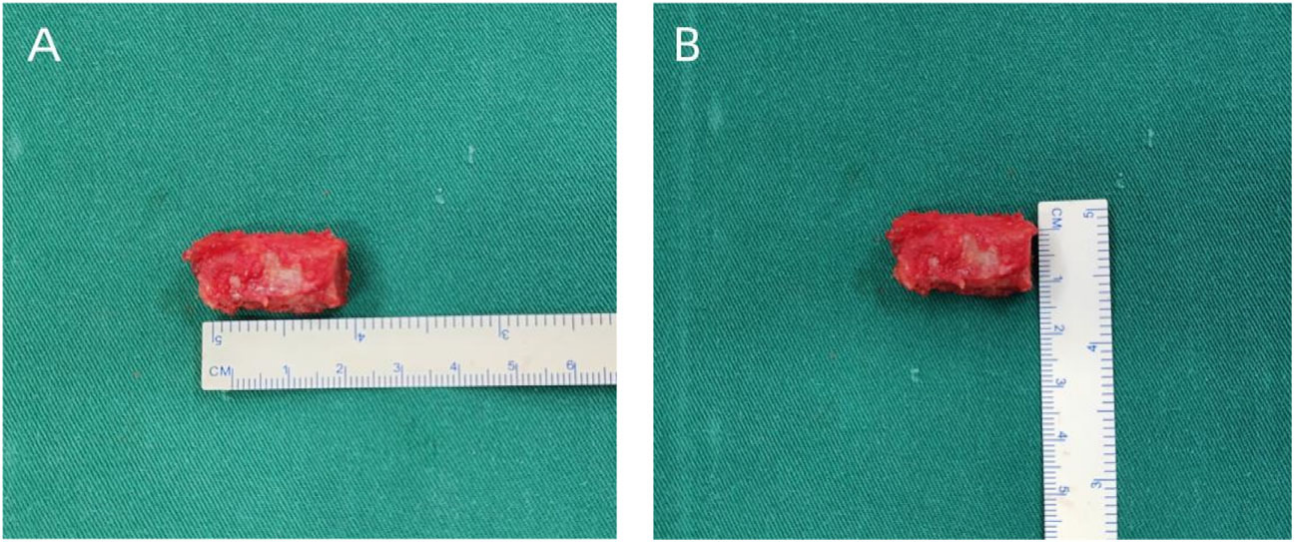
Iliac crest graft. The sizes of the block were 2 cm long (A) × 1 cm high × 1 cm wide (B).

An approximately 2‐cm oblique longitudinal skin incision is made 3 cm posterior to the anterior superior iliac spine on the ipsilateral side (Figure 3). The subcutaneous tissue is dissected down to the bony surface, carefully preserving the integrity of the external iliac cortex. Using an oscillating saw, a rectangular bone block graft is harvested. The sizes of the block are 2 cm long × 1 cm high × 1 cm wide (based on the preoperative 3‐dimensional printing of the glenoid) (Figure 4).

**FIGURE 5:**
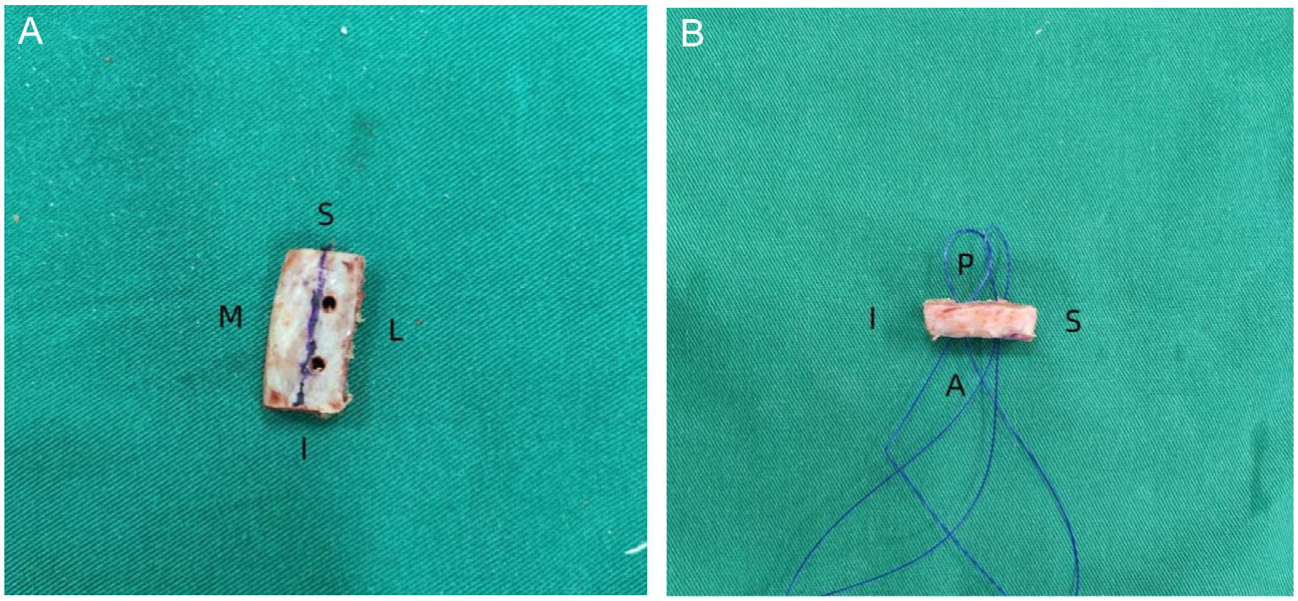
Prepped iliac crest allograft. (A) Two holes were drilled at one‐third and two‐thirds points along the longitudinal axis of the bone block, slightly offset from the midline. (B) Two folded polydioxanone sutures were passed through these holes. (A, anterior; I, inferior, L, lateral; M, medial; P, posterior; S, superior.)

The bone block is then trimmed to optimize the fit of its cancellous surface against the prepared glenoid defect. Two holes are drilled at one‐third and two‐thirds points along the longitudinal axis of the bone block, slightly offset from the midline. Two folded polydioxanone sutures are passed through these holes to facilitate subsequent bone block fixation (Figure 5).

### Preparation of the Glenoid Bone Defect

**FIGURE 6:**
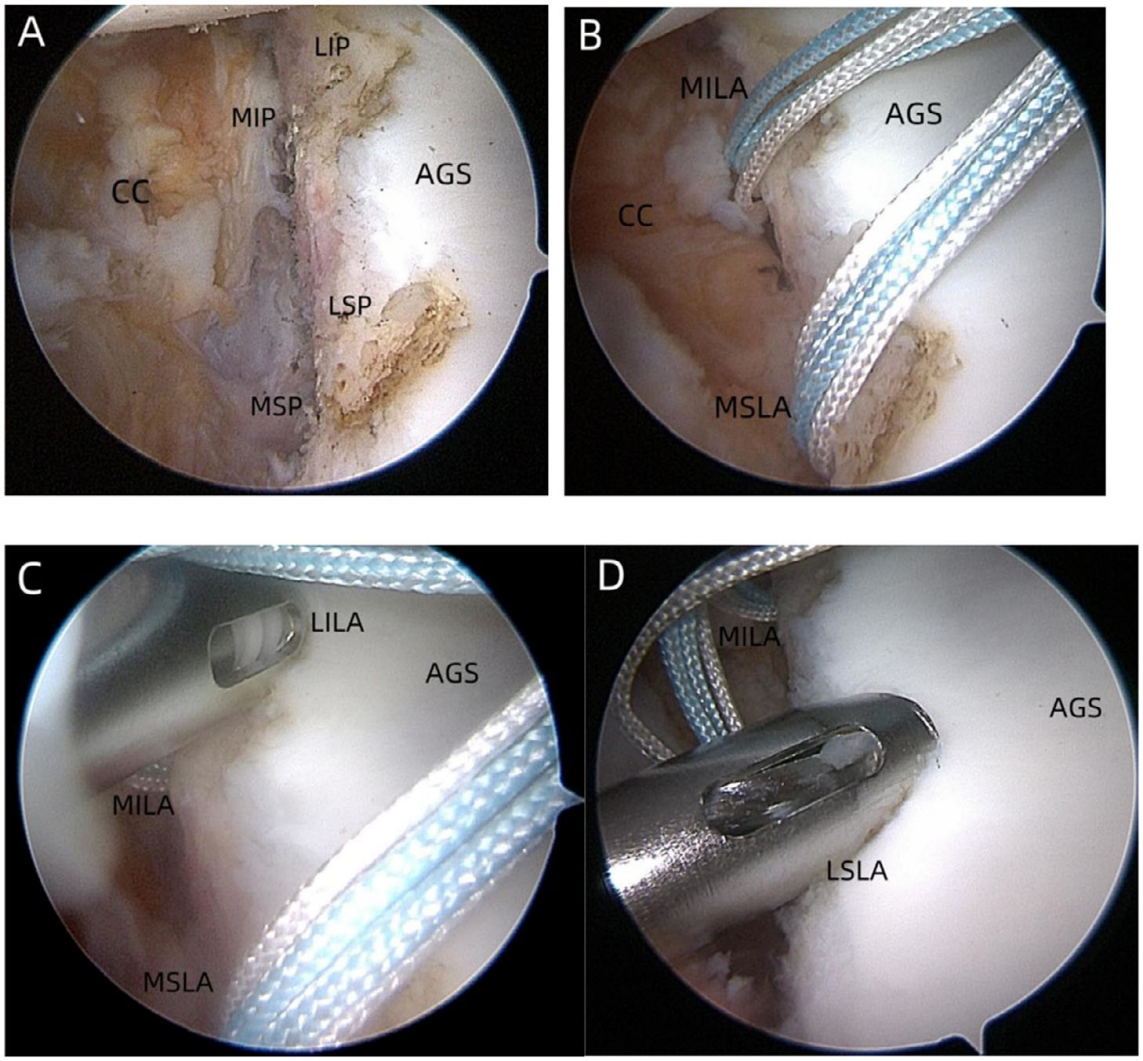
Right shoulder. Arthroscopic view, anterior portal. (A) The labrum anchor positions were marked by plasma. (B) The medial labrum anchors were inserted at the marked position. (C) The lateral inferior labrum anchor was inserted. (D) The lateral superior labrum anchor was inserted. (AGS, anterior glenoid surface; CC, capsulolabral complex; LILA, the lateral inferior labrum anchor; LIP, the lateral inferior point; LSLA, the lateral superior labrum anchor; LSP, the lateral superior point; MILA, the medial inferior labrum anchor; MIP, the medial inferior point; MSLA, the medial superior labrum anchor; MSP, the medial superior point.)

Through the anterior portal, establish an anterosuperolateral portal and use a grinding head (Star, Beijing, China) to polish the defect of the glenoid bone for freshness through it. Then, mark the one‐third and two‐thirds points along the longitudinal axis of the glenoid bone defect by a radiofrequency plasma knife (Star, Beijing, China), slightly offset from the midline and corresponding the superior and inferior lateral edges of it (Figure 6A). Insert a 3.0‐mm PEEK labrum anchor (FixTree, Star, Beijing, China) at each of the 4 points through the anterior portal (Figure 6B‐D).

### Insertion and Fixation of the Bone Graft

**FIGURE 7:**
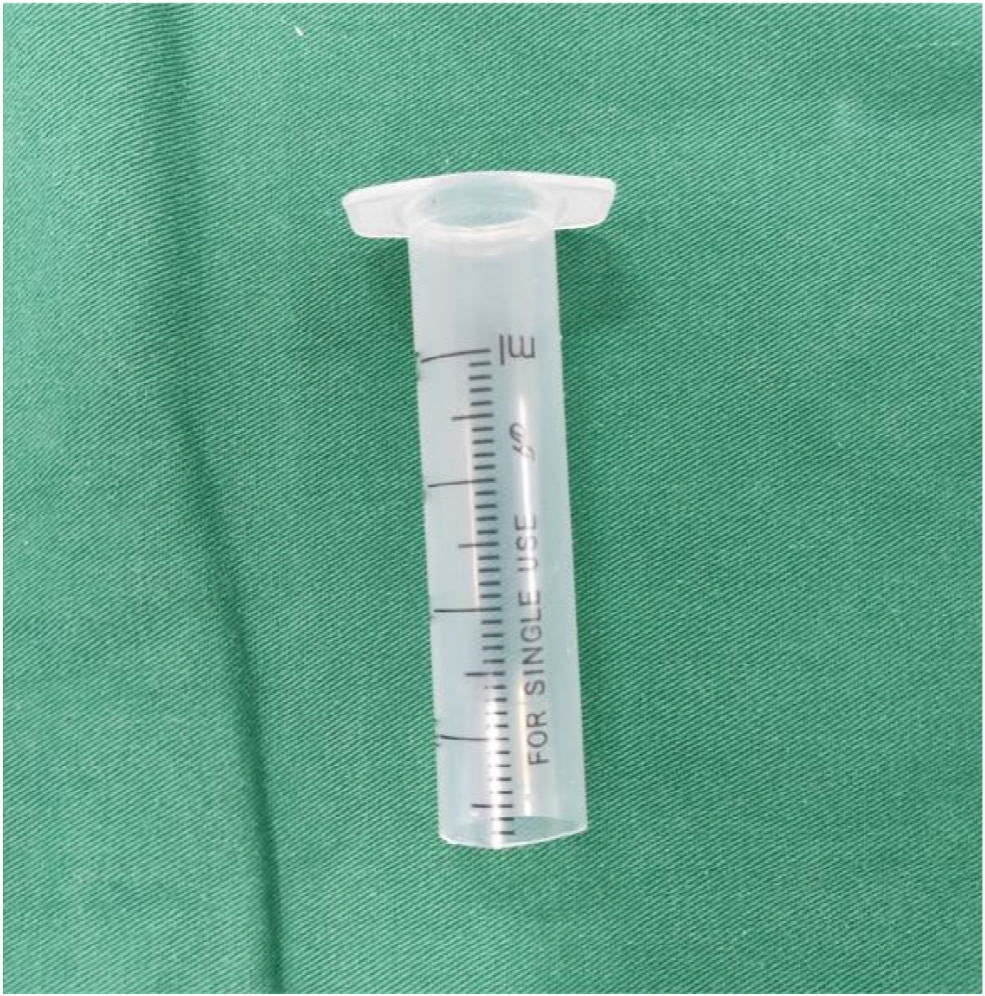
Photograph of a head‐removed 10 mL syringe.

**FIGURE 8:**
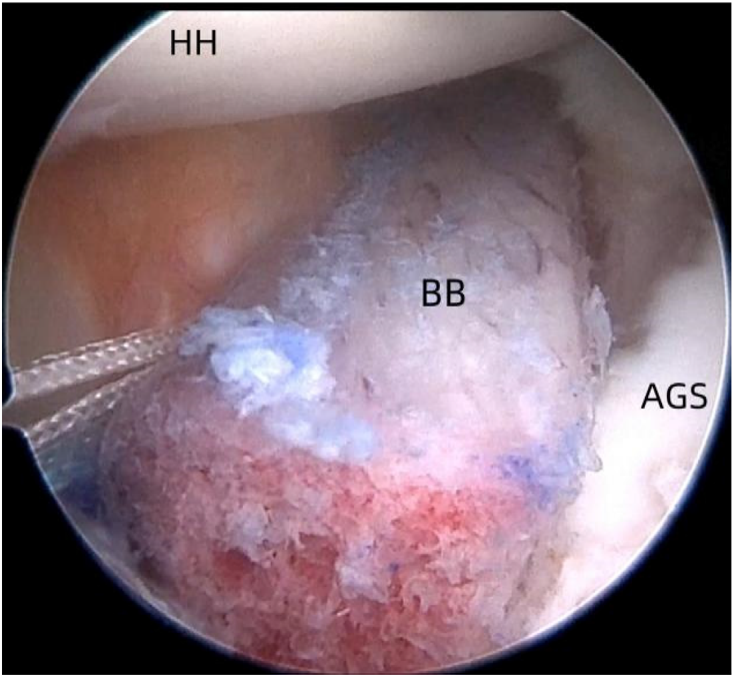
Right shoulder. Arthroscopic view, anterior portal. The bone block was sent into the joint through the anterior portal. (AGS, anterior glenoid surface; BB, bone block; HH, humeral head.)

**FIGURE 9:**
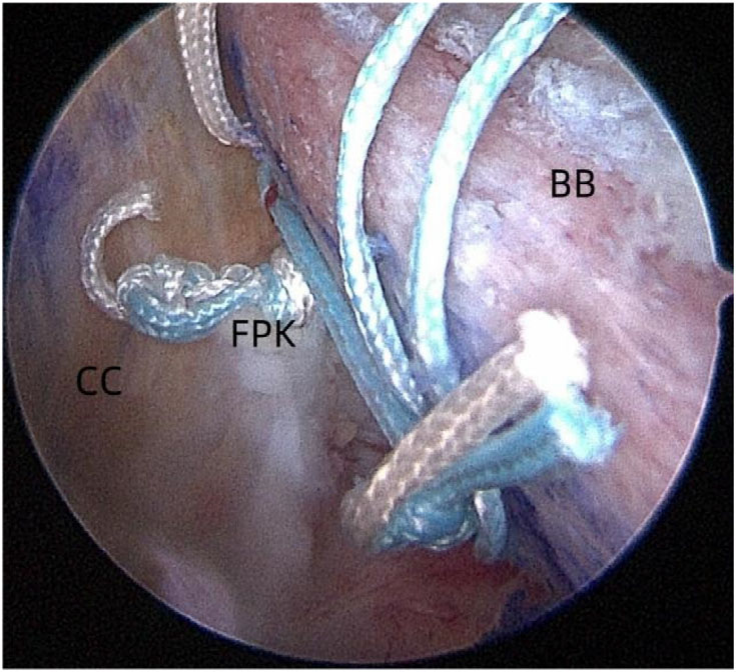
Right shoulder. Arthroscopic view, anterior portal. The first pulley knot on 2 limbs for each of the 2 medial labrum anchors was made. (BB, bone block; CC, capsulolabral complex; FPK, first pulley knot.)

**FIGURE 10:**
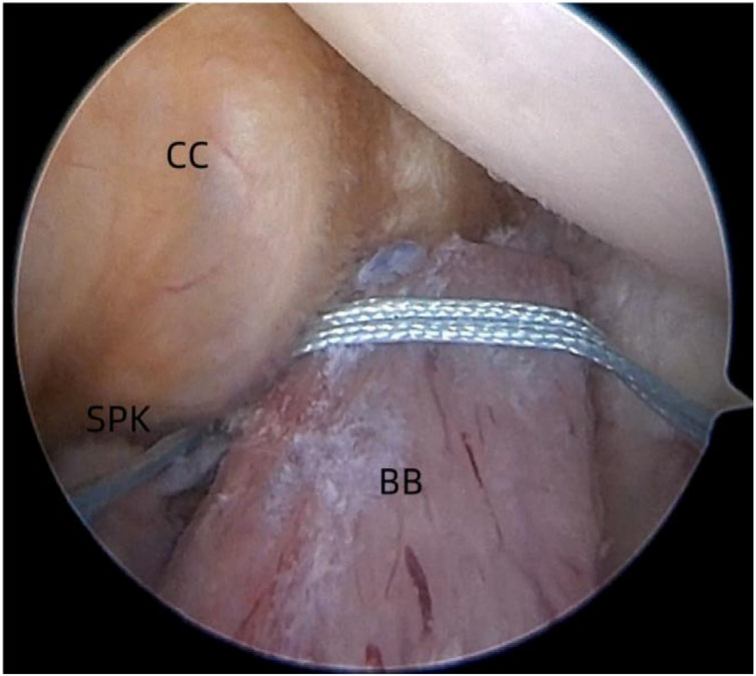
Right shoulder. Arthroscopic view, anterior portal. The second pulley knot was tied on the remaining limbs of the medial superior labrum anchor and 2 limbs of the lateral superior labrum anchor. (BB, bone block; CC, capsulolabral complex; SPK, second pulley knot.)

**FIGURE 11:**
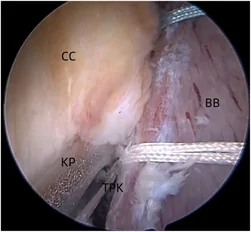
Right shoulder. Arthroscopic view, anterior portal. The third pulley knot was made by the other limbs of the medial inferior labrum anchor and 2 limbs of the lateral inferior labrum anchor. (BB, bone block; CC, capsulolabral complex; KP, knot pusher; TPK, third pulley knot.)

**FIGURE 12:**
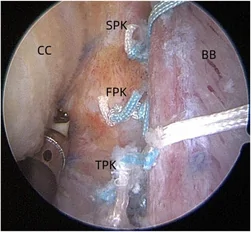
Right shoulder. Arthroscopic view, anterior portal. The “3‐pulley 4‐point antirotation” was formed to stabilize the bone block. (BB, bone block; CC, capsulolabral complex; FPK, first pulley knot; SPK, second pulley knot; TPK, third pulley knot.)

Insert a head‐removed 10 mL syringe (Figure 7) through the anterosuperolateral portal into the glenohumeral joint as a graft channel. Using the folded polydioxanone sutures, the 4 limbs of 2 medial labrum anchors are introduced into the reserved holes. Through this channel and labrum anchors sutures, the bone block is sent into the joint (Figure 8). Next, adjust the position of the bone block by a basket clamp until it fits against the glenoid defects. Use a wire clamp to perform the first pulley knot on 2 limbs for each of the 2 medial labrum anchors to stabilize the bone block (Figure 9). A second pulley knot is tied on the remaining limbs of the medial inferior labrum anchor and 2 limbs of the lateral inferior labrum anchor (Figure 10). Afterward, the third pulley knot is made by the other limbs of the medial superior labrum anchor and 2 limbs of the lateral superior labrum anchor (Figure 11). In this way, a “3‐pulley 4‐point antirotation” is formed to stabilize the bone block (Figure 12).

### Labral Repair

**FIGURE 13:**
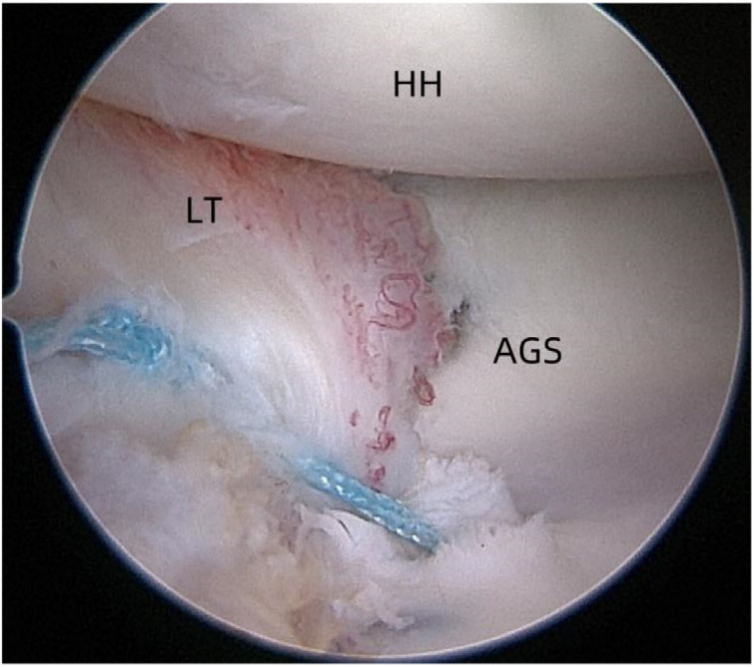
Right shoulder. Arthroscopic view, anterior portal. Repair of bankart lesion. (AGS, anterior glenoid surface; HH, humeral head; LT, labrum tissue.)

**FIGURE 14:**
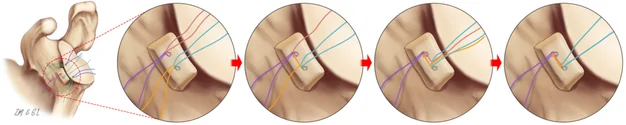
Schematic drawing of the process of making a pulley knot.

**FIGURE 15:**
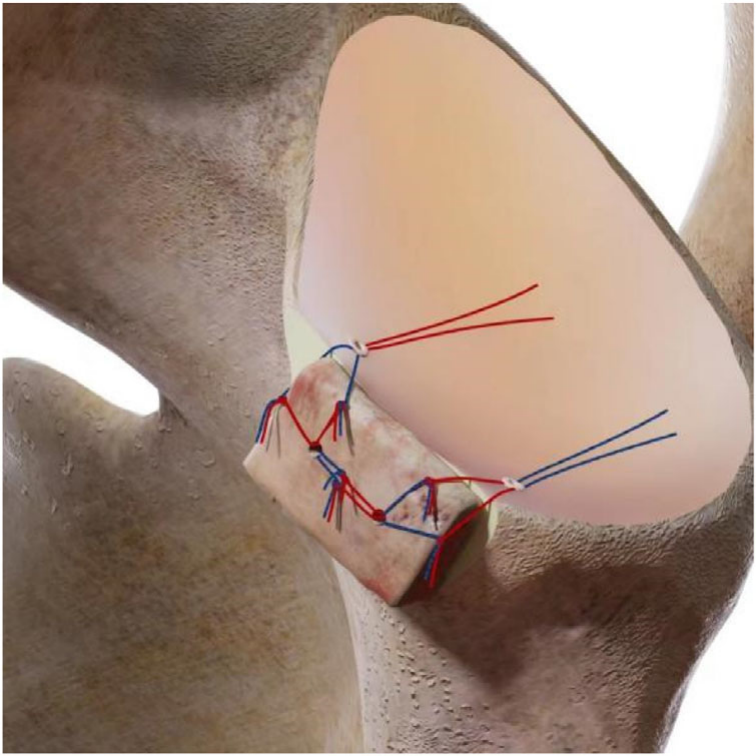
Schematic drawing of autologous iliac bone grafting with “3‐pulley 4‐point antirotation suture anchor technique” for the treatment of recurrent anterior shoulder dislocation.

The remaining limbs of 2 lateral labrum anchors are shuttled through the labral tissue for bankart repair through the anterosuperolateral portal, thereby further stabilizing the bone block (Figure 13). There are simplified schematic diagrams of the technology (Figures 14 and 15). Some tips and pitfalls of the technique are described in Table 2.

**TABLE 2:** Pearls and Pitfalls

Pearls
• Bone blocks are harvested from the medial plate of the iliac bone on the same side of the affected shoulder
• The 2 reserved holes position is more inclined toward the inner side of the midline of the bone block to increase the load‐bearing area and enhance stability
• Freshen the glenoid bone defect and adhere the cancellous surface of the grafts to it
• During the process of the graft implantation, the fitting surface always corresponds to the glenoid surface, and the tension of the medial labrum anchor sutures is maintained to prevent the bone block from flipping over
• Wrap the labrum and soft tissue around the bone block as much as possible to provide rich blood supply for the bone block and promote its healing

Pitfalls
• The position of the anchors should be accurately marked to avoid graft malpositioning
• The direction of anchor insertion should be vertical as much as possible to the glenoid surface to avoid anchor detachment
• It is important to make the first pulley knot with the inner anchor limbs to stabilize the bone block and then make the remaining pulley knots to avoid graft malpositioning
• The fixation force of the bone block is moderate, and its movement should be within 1 mm after fixation is completed
• Repair bankart injuries as much as possible, minimize damage to the labrum and soft tissues, and maintain their integrity to enhance bone stability

### Remplissage

Preoperative 3‐dimensional reconstruction of the humeral head shows the off‐track lesion of the humeral head defect, and the additional remplissage procedure is performed. Through a posterior portal, 2 4.5‐mm PEEK suture anchors (FastLock, Star, Beijing, China) are inserted into the posterior superior defect of the humeral head, with the sutures shuttling through the infraspinatus, teres minor, and soft tissues. Then, the sutures were knotted.

### Rehabilitation

The patients are immobilized with an abduction brace for 6 weeks after surgery. A passive range of motion exercises such as forward bend and upward lift, abduction, and internal and external rotation of the affected shoulder is started 2 days after surgery, with the range of motion based on the patient's maximum tolerance for pain. After 6 weeks, remove the brace and start active shoulder flexion, elevation, abduction, and internal and external rotation activities. Start strength training for the affected shoulder after 3 months, including elbow flexion resistance and shoulder internal and external rotation resistance training. Engage in adversarial sports, throwing exercises, and heavy physical activities after 6 months.

## DISCUSSION

Currently, the main fixation methods of arthroscopic bone grafting are screw and suture button. Some studies show that complications of screw fixation include bone resorption, screw failure, migration of bone block and bone nonunion. The incidence of screw failure (screw loosening, bending, and breakage) is 3.8% to 35%.^14^, ^15^, ^16^ If screw failure occurs, the handling of screws during a second revision surgery can be a challenging issue. There is a certain relation between long‐term postoperative shoulder joint pain and screw irritation. Other studies showed that the dissolution and absorption of bone blocks could cause screw protrusion, leading to tear of the subscapularis muscle, humeral head impingement, and the formation of osteoarthritis, which were the main causes of shoulder joint pain.^17^ In addition, the insertion of screws under the arthroscope needs to be perpendicular to the graft and glenoid bone, which is difficult and has a long learning curve. Furthermore, the rate of failure of screws that are too short or those that inserted at an angle greater than 25° to the surface of the glenoid joint has increased significantly.^18^ Suture button fixation can effectively avoid the problems of humeral head impact and arthritis formation caused by screw fixation and does less damage to the bone, with satisfactory clinical efficacy.^7^, ^9^ However, its fixation force may be weaker than that of the screw. Williams et al.^19^ compared the failure load and bone displacement of 2 fixation methods on shoulder joint specimens, and the results showed that screw fixation was superior to suture button. Bonnevialle et al.^20^ reported that the bone healing rates of suture button and screw fixation were 41% and 100%, respectively, 3 months after surgery, and the bone contact area of suture button was lower than that of screw. Besides, we believe that although the internal plant does not need to be removed, the retention of the suture button in the joint is also a possible pitfalls. The suture button belongs to the linear fixation in the sagittal plane, which may result in insufficient antirotation force in the coronal plane.

Anchor suture fixation of bone blocks can avoid the problems caused by screw and suture button. Because of its elastic fixation, it causes less damage to the grafts and can reduce the absorption rate of bone blocks. However, Ritter et al. reported that suture anchor fixation has issues of insufficient force and easy rotation.^13^ This technique we introduced in this article can improve the fixation strength of the autogenous iliac bone block by inserting 4 labrum suture anchors and using pulley knot for fixation. Clinical application showed that, at the last follow‐up, all patients did not experience serious complications. The imaging results showed that there was no displacement in the position of the bone blocks, and all bone blocks achieved bony healing with partial absorption and remodeling.

To summarize, arthroscopic autologous iliac bone grafting with the “3‐pulley 4‐point antirotation suture anchor technique” for the treatment of recurrent anterior shoulder dislocation can effectively prevent bone block rotation, can provide more reliable fixation, achieve good clinical efficacy, and is easy to operate. Future research should focus on biomechanical outcomes to show the reliability of this technique.

## DISCLOSURES

The authors (L.G., H.H., W.Z., S.F., L.H., M.Z.) declares that they have no known competing financial interests or personal relationships that could have appeared to influence the work reported in this article.
